# Genome-Wide Analysis of Respiratory Burst Oxidase Homologs in Grape (*Vitis vinifera* L.)

**DOI:** 10.3390/ijms141224169

**Published:** 2013-12-12

**Authors:** Chenxia Cheng, Xiaozhao Xu, Min Gao, Jun Li, Chunlei Guo, Junyang Song, Xiping Wang

**Affiliations:** 1 State Key Laboratory of Crop Stress Biology in Arid Areas, College of Horticulture, Northwest A&F University, Yangling, Shaanxi 712100, China; E-Mails: chengchenxia@163.com (C.C.); xxz@nwsuaf.edu.cn (X.X.); gaomin.001@163.com (M.G.); junli2011050448@gmail.com (J.L.); guo0208chun@163.com (C.G.); songjunyang@nwsuaf.edu.cn (J.S.); 2 Key Laboratory of Horticultural Plant Biology and Germplasm Innovation in Northwest China, Ministry of Agriculture, Northwest A&F University, Yangling, Shaanxi 712100, China

**Keywords:** reactive oxygen species, synteny analysis, phylogenetic analysis, gene expression

## Abstract

Plant respiratory burst oxidase homolog (*rboh*) genes appear to play crucial roles in plant development, defense reactions and hormone signaling. In this study, a total of seven *rboh* genes from grape were identified and characterized. Genomic structure and predicted protein sequence analysis indicated that the sequences of plant *rboh* genes are highly conserved. Synteny analysis demonstrated that several *Vvrboh* genes were found in corresponding syntenic blocks of *Arabidopsis*, suggesting that these genes arose before the divergence of the respective lineages. The expression pattern of *Vvrboh* genes in different tissues was assessed by qRT-PCR and two were constitutively expressed in all tissues tested. The expression profiles were similarly analyzed following exposure to various stresses and hormone treatments. It was shown that the expression levels of *VvrbohA*, *VvrbohB* and *VvrbohC1* were significantly increased by salt and drought treatments. *VvrbohB*, *VvrbohC2*, and *VvrbohD* exhibited a dramatic up-regulation after powdery mildew (*Uncinula necator* (Schw.) Burr.) inoculation, while *VvrbohH* was down-regulated. Finally, salicylic acid treatment strongly stimulated the expression of *VvrbohD* and *VvrbohH*, while abscisic acid treatment induced the expression of *VvrbohB* and *VvrbohH*. These results demonstrate that the expression patterns of grape *rboh* genes exhibit diverse and complex stress-response expression signatures.

## 1. Introduction

Reactive oxygen species (ROS) play multiple signaling roles in a wide range of organisms, including bacteria and mammals, and are also known to control various cellular mechanisms in plants. Indeed, there is growing evidence that ROS are key to fundamental plant metabolic processes, such as cellular growth [1,2], the hypersensitive response (HR) and abiotic stress responses [3–5]. Furthermore, ROS are integrated into many different signaling systems in plants, such as those mediated by protein kinases, calcium and hormones [6].

The major source of ROS in plants is the NADPH oxidase-catalyzed conversion of the superoxide anion (O_2_·^−^) to other ROS, such as perhydroxyl radicals, hydroxyl radicals and hydrogen peroxide [7]. The *respiratory burst oxidase homolog* (*rboh*) gene family encodes the key enzymatic subunit of the plant NADPH oxidase, the first example of which to be identified was the rice *rbohA* gene, which is a homologue of the mammalian gene *gp91^phox^* [8]. Following this initial discovery, *rboh* genes have been identified from other plant species, including *Arabidopsis thaliana* [9], tomato [10,11], tobacco [12–15], potato [16,17], maize [18], watermelon [19], barley [20,21], *Medicago truncatula* [22] and *Lepidium sativum* [23]. The proteins predicted to be encoded by the mammalian *gp91^phox^* gene and homologous plant *rboh* genes share conserved structural and functional domains, but the plant sequences differ in that they have an extended *N*-terminal region. This extension contains two putative calcium-binding domains (EF-hands), which may account for their direct regulation by Ca^2+^[3].

The *rboh* gene family has been most extensively characterized in *Arabidopsis*, where members play crucial roles in plant health and metabolism. Sagi and Fluhr [24] identified 10 *rboh* homologs in the *Arabidopsis* genome and it has been shown by microarray analysis that *AtrbohH* and *AtrbohJ* are specifically expressed in pollen, *AtrbohA-G* and *AtrbohI* are specifically expressed in roots, while *AtrbohD* and *AtrbohF* are expressed in all plant tissues (https://www.genevestigator.com) [25]. *AtrbohD* and *AtrbohF* participate in guard cell ABA signal transduction [26] and are also required for the accumulation of reactive oxygen intermediates during plant defense responses [27]. Transient RNA interference-mediated gene silencing of barley *HvrbohA* indicated a potential role in influencing penetration by the powdery mildew fungus *Blumeria graminis* f. sp. *Hordei* [20]. Moreover, the tobacco *NtrbohD* gene is responsible for ROS production in cryptogein-elicited tobacco cells [15], and *NbrbohA* and *NbrbohB* participate in H_2_O_2_ accumulation and resistance to *Phytophthora infestans* [14]. In addition, the *Arabidopsis* NADPH oxidase *rbohD* mediates rapid, long-distance, cell-to-cell signaling, which can be triggered by diverse stimuli, including wounding, heat, cold, high-intensity light and salinity stresses [28]. Together, these results indicate an important role for *rboh* genes in plant stress responses; however, there is also evidence that they also function in the regulation of plant growth and development. The *Arabidopsis rhd2* mutant, which lacks a functional *AtrbohC* gene is defective in root hair growth, and it has been suggested that the corresponding protein affects ROS-mediated plant cell growth through the activation of Ca^2+^ channels [2]. In addition, cress plants in which *LesarbohB* expression was suppressed showed a strong seedling root phenotype that resembles those associated with defective auxin-related genes, thus indicating that *LesarbohB* plays a role in root development via auxin signaling [23].

To our knowledge, a functional analysis of grapevine (*Vitis vinifera* L.) *Vvrboh* genes has not yet been reported. Grapevine is one of the most important perennial fruit crops worldwide and the release of the grape genome data now allows a comprehensive genome-wide identification and analysis of *Vvrboh* genes. We also report an analysis of exon-intron structure, phylogenetic relationships and synteny with the *Arabidopsis rboh* gene family. Finally, the expression profiles of *Vvrboh* genes in different tissues, and in grape leaves responding to different exogenous hormones as well as abiotic and biotic stresses are presented; data that we propose will provide a solid foundation for future functional analyses.

## 2. Results

### 2.1. Identification of *Vvrboh* Genes in the Grape Genome

A total of seven genes from the grape genome were predicated to encode Rboh proteins (Table 1), and according to their localization in the grape genome and the widely recognized nomenclature [22] were named *VvrbohA*, *VvrbohB*, *VvrbohC1*, *VvrbohC2*, *VvrbohD*, *VvrbohE* and *VvrbohH*. All seven genes were mapped to a specific chromosome (1, 2, 6, 11, 14 and 19). The smallest predicted gene length was that of *VvrbohH* (3707 bp) and the largest was that of *VvrbohC2* (15,930 bp), while their predicted open reading frames were similar in length. Computational prediction of protein localization indicated that VvRbohA, VvRbohC1 and VvRbohD are localized in the plasma membrane while the other Rbohs were predicted to reside in the chloroplast thylakoid membrane.

### 2.2. Sequence Analysis and Domain Organization in Grape Rboh Homologs

**Figure 1.**
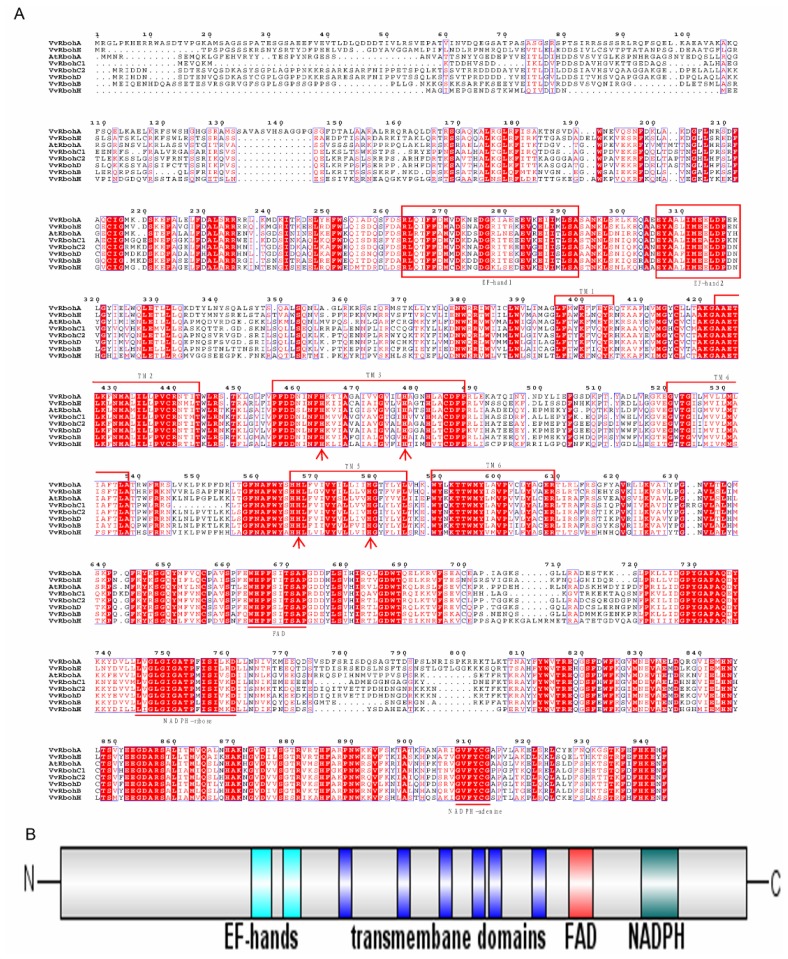
Protein alignment and domain structure of AtRbohA and the seven predicted grape respiratory burst oxidase homolog (Rboh) proteins. (**A**) The red shading indicates residues that are identical and the lighter shading represents positions with a lower level of conservation. EF-hand domains are indicated by the boxes. Conserved binding sites for flavin adenine dinucleotide (FAD), NADPH-ribose and NADPH-adenine are indicated with a straight line below the alignment. Histidine residues involved in heme binding are indicated by arrows. Putative transmembrane domains (TMs) are indicated by brackets above the alignment; and (**B**) Schematic representation of the grape Rboh proteins with their respective functional domains, showing that grape Rboh proteins are similar to *Arabidopsis* AtRbohA.

The amino acid sequences of the grapevine Rboh homologs were aligned with *Arabidopsis* AtRbohA to identify conserved domains, including those for binding of FAD, NADPH-ribose and NADPH-adenine in the *C*-terminal region (Figure 1A,B) [29,30]. The *N*-terminal regions of the seven predicted VvRboh proteins each contain two putative Ca^2+^-binding EF-hands, which are known to play a key role in the regulation of Rbohs [7,31,32] and were predicted to include six transmembrane domains (TM1–6) that correspond to those identified in plant Rbohs from *Arabidopsis*, rice, maize, barley, potato and tobacco, as well as the mammalian gp91^phox^. TM3 and TM5 also contain pairs of histidine residues that have been reported to be important for heme binding in the human gp91^phox^ protein during electron transfer across the cell membrane [33].

### 2.3. Phylogenetic Analysis of *Vvrboh* Genes

**Figure 2.**
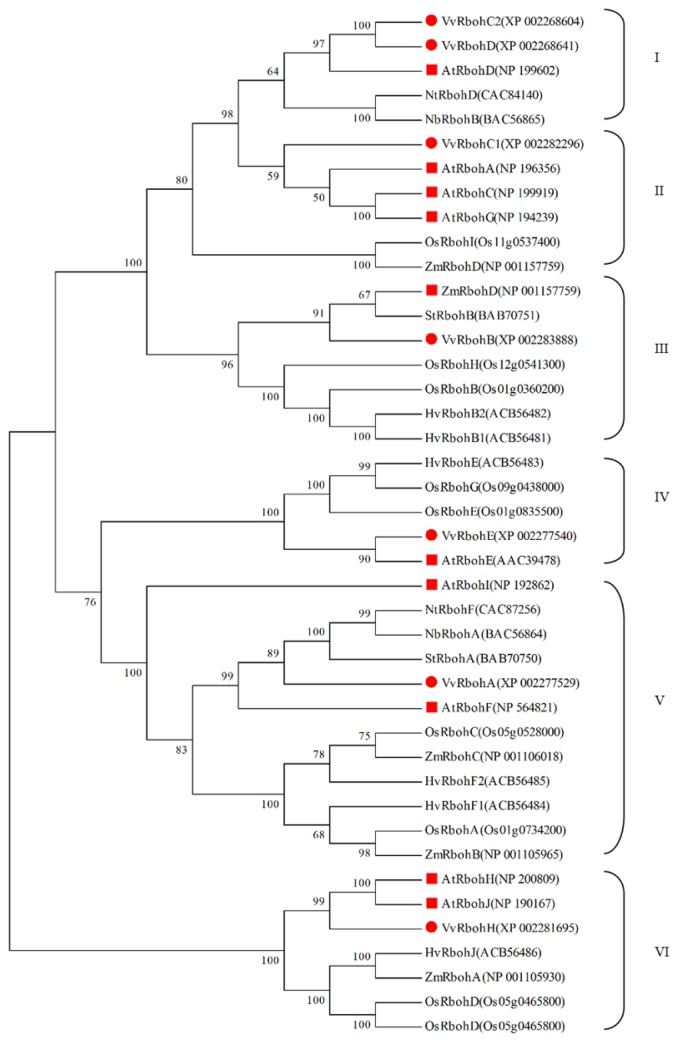
Phylogenetic analysis of grape and other plant Rboh proteins. The phylogenetic tree was constructed with Rboh domain protein sequences from *V. vinifera* (VvRboh), *N. tabacum* (NtRboh), *Z. mays* (ZmRboh), *O. sativa* (OsRboh), *A. thaliana* (AtRboh), *S. tuberosum* (StRboh), *N. benthamiana* (NbRboh) and *H. vulgare* (HvRboh). They were classified to six groups: I, II, III, IV, V, VI. VvRboh proteins are indicated with red circles and AtRboh proteins with red boxes. All accession numbers or locus IDs of the *rboh* genes are listed in the phylogenetic tree.

In order to infer the evolutionary relationships among plant Rbohs, the predicted VvRboh amino acid sequences were compared with each other and with divergent VvRboh from *Arabidopsis*, *N. tabacum*, *Z. mays*, *O. sativa*, *S. tuberosum*, *N. benthamiana* and *H. vulgare* [21]. Six groups of orthologs were identified (Figure 2), which is in agreement with previous evolutionary analyses of plant Rbohs [34]. The VvRboh sequences were distributed amongst all groups (Figure 2): VvRbohA, VvRbohB, VvRbohC1, VvRbohE and VvRbohH belong to group V, III, II, IV and VI, respectively, while VvRbohC2 and VvRbohD belong to group I.

### 2.4. Gene Structure Analysis of *Vvrboh* Genes

**Figure 3.**
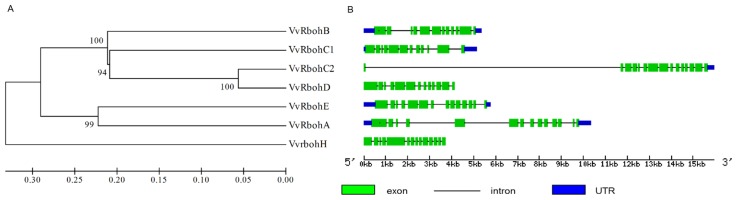
Phylogenetic analysis (**A**) and exon-intron structures (**B**) of *Vvrboh* genes. Numbers above or below branches in the tree indicate bootstrap values. Exons, introns and untranslated regions (UTR) are indicated by green boxes, black horizontal lines and blue boxes, respectively.

An unrooted phylogenetic tree was constructed using only the VvRboh protein sequences identified in this study (Figure 3A). Exon-intron structures were identified based on the coding sequences and the corresponding genome sequences (Figure 3B) and the similarity of the grapevine genes to those previously described in *Arabidopsis*, barley and rice was further reflected in the intron/exon structures. Within their coding regions *HvrbohB1*, *HvrbohB2* and *VvrbohC2* contain 12 exons, compared to the 13 exons of *HvrbohE*, *HvrbohF2*, *VvrbohC1* and *VvrbohE*, the 14 exons of *AtrbohF*, *OsrbohA*, *HvrbohF1*, *HvrbohJ*, *VvrbohA* and *VvrbohH* and the 15 exons of *VvrbohB* and *VvrbohD*. The increase in exon number seen in several of the grapevine genes appears to be a result of insertions of introns into the exonic regions, rather than from acquisition of additional exons. The order and approximate size of exons among the *Vvrboh* genes is relatively conserved, while intron size is more variable. Spacing between the first and second, as well as between the eleventh and twelfth exon is particularly variable, as seen in the first exon of *VvrbohB*, *VvrbohC1* and *VvrbohD*, which is different from those in the other genes and might reflect a division of the first exon into two or three exons during evolution.

### 2.5. Evolutionary Relationships between Grape and *Arabidopsis rboh* Genes

**Figure 4.**
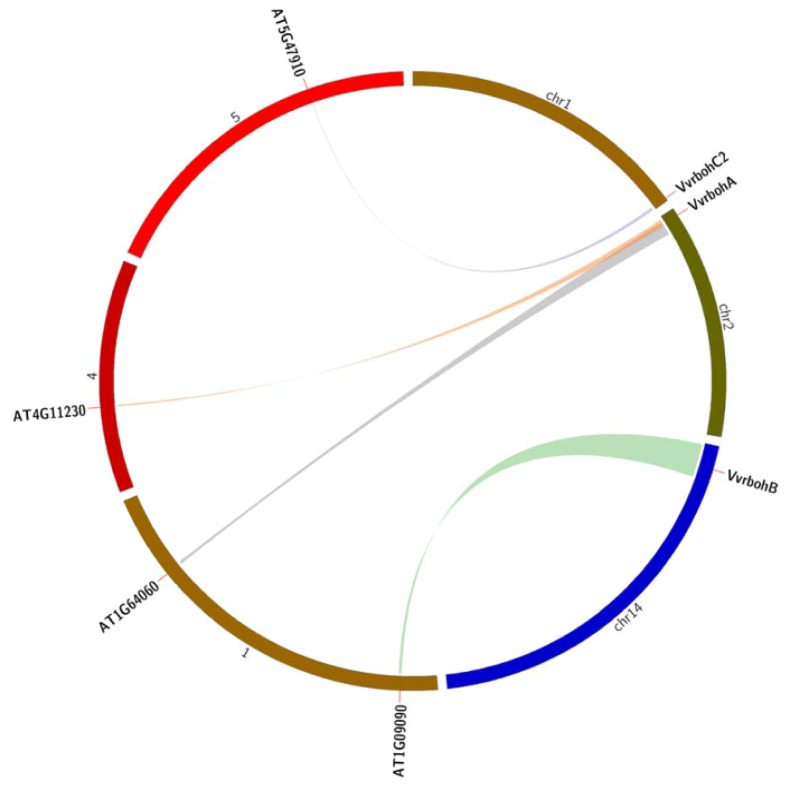
Synteny analysis of *rboh* genes from grape and *Arabidopsis.* The positions of related *Vvrboh* genes and *Atrboh* genes are depicted in the grape chromosomes (chr1, 2 and 14) and *Arabidopsis* chromosomes (1, 4 and 5), respectively. Colored lines connecting two chromosomal regions indicate syntenic regions between grape and *Arabidopsis* chromosomes.

*A. thaliana* is one of the most important experimental model plant species and the functions of most *Arabidopsis rboh* genes have been well characterized. Accordingly, a comparative syntenic map between grape and *Arabidopsis* genomes was created in order to further study the origin, evolutionary history and putative functions of the grape homologs. Syntenic groups containing orthologs of three *Vvrboh* genes and four *rboh* genes from *Arabidopsis* were identified (Figure 4). According to this analysis, four paired *Vvrboh*-*Atrboh* genes (*VvrbohA*-*AT4G11230*, *VvrbohA*-*AT1G64060*, *VvrbohB*-*AT1G09090* and *VvrbohC2*-*AT5G47910*) were located in genomic regions with synteny between the grape and *Arabidopsis* genomes (Table S1), indicating that these genes may be derived from a common ancestor. Based on this type of comparative genomic analysis, it is possible to deduce potential function of genes to guide future functional studies.

### 2.6. Expression Patterns of *Vvrboh* Genes in Different Tissues and Organs

**Figure 5.**
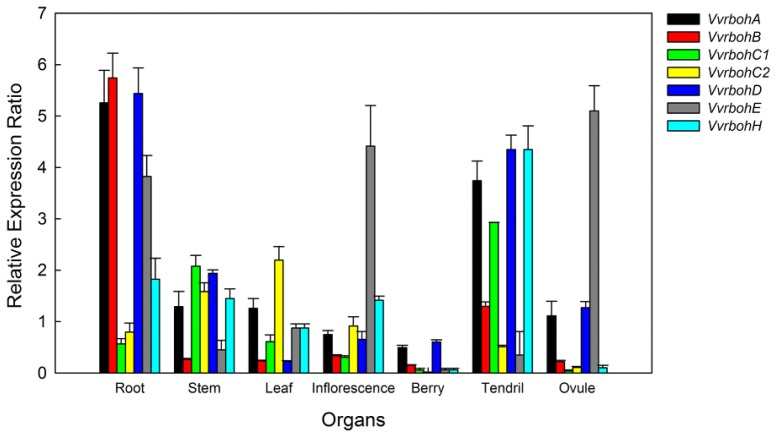
The expression profiles of seven *Vvrboh* genes in various tissues. qRT-PCR analysis was conducted to visualize *Vvrboh* gene expression. Amplification of *actin1* was used as an internal control. Error bars represent standard error (SE; *n* = 3).

qRT-PCR was performed using RNA isolated from young leaves, roots, stems, inflorescences, berries, tendrils and ovules, revealing differential expression patterns for the seven *Vvrboh* genes (Figure 5). *VvrbohA*, *VvrbohB*, *VvrbohD* and *VvrbohH* were more highly expressed in roots and tendrils and *VvrbohB*, *VvrbohC1*, *VvrbohC2* and *VvrbohH* had much lower expression in the ovules and berries. *VvrbohE* transcripts were more abundant in roots, inflorescences and ovules compared to other tissues/organs tested.

### 2.7. Expression Profiles of *Vvrboh* Genes Following Various Stress Hormone Treatments

**Figure 6.**
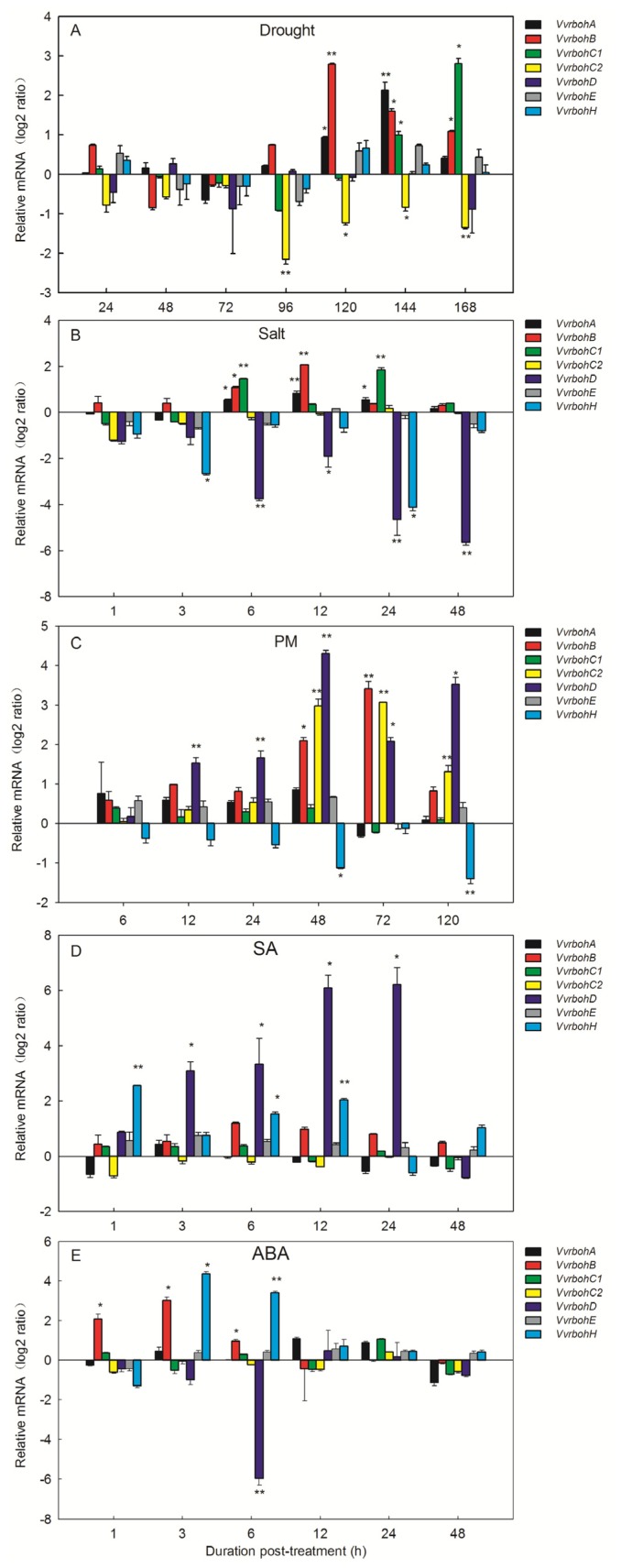
Effect of drought (**A**); salt (**B**); powdery mildew (PM) (**C**); salicylic acid (SA) (**D**) and abscisic acid (**E**) on the expression of *Vvrboh* gene expression in leaves was investigated by qRT-PCR. The grape *actin1* gene was used as the reference gene. Error bars represent SE (*n* = 3). Asterisks indicate levels of significance of differential expression (*t-*test, *p ≤ 0.05, **p ≤ 0.01).

In order to determine whether the *Vvrboh* genes responded to stress conditions, we examined their expression patterns in response to a series of stress and exogenous hormone treatments. To test abiotic stress effects, drought and salinity treatments were performed. As shown in Figure 6A,B, the expression levels of *VvrbohA*, *VvrbohB* and *VvrbohC1* were significantly increased by salt and drought treatments, while *VvrbohC2* was down-regulated under drought treatment. Only the transcripts of *VvrbohD* and *VvrbohH* were down-regulated by the salt treatment, while *VvrbohE* expression was not altered by either treatment.

Powdery mildew was used to infect grapevine to test for responses to biotic stress. *VvrbohB*, *VvrbohC2*, and *VvrbohD* exhibited a dramatic up-regulation after powdery mildew inoculation, while *VvrbohH* was down-regulated (Figure 6C). The expression of the remaining *Vvrboh* genes did not change appreciably after inoculation with powdery mildew. Responses to exogenous hormone treatments were also evaluated by spraying grape leaves with either SA or ABA. After SA treatment (Figure 6D), *VvrbohA*, *VvrbohB*, *VvrbohC1*, *VvrbohC2* and *VvrbohE* showed a constitutive expression pattern, while *VvrbohD* and *VvrbohH* were significantly induced. As shown in Figure 6E, *VvrbohD* showed obvious decreases in expression 6 h after ABA treatment while *VvrbohB* and *VvrbohH* showed an increase in expression at 1 to 6 h post-treatment. All the remaining *Vvrboh* genes showed no appreciable changes in transcript levels following ABA treatment, and the expression level of *VvrbohE* did not change appreciably under any tested condition.

## 3. Discussion

### 3.1. Identification and Sequence Analysis of *Vvrboh* Genes

In this study we identified seven grape *rboh* genes (Table 1), of which *VvrbohA*, *VvrbohC1* and *VvrbohD* were predicted to encode proteins that are located in the plasma membrane, suggesting that their functions are similar to those of other plant homologs. In contrast to *Arabidopsis* and rice, where Rbohs are predicted to localize to the plasma membrane, VvRbohB, VvRbohC2, VvRbohE and VvRbohH were predicted to be located in the thylakoid membrane of the chloroplast, indicating other functions. We are interested in the potential connection between cellular localization and functionalities of the Vvrboh protein and further experimental analyses are being carried out to try to analyze it. A comparison of the predicted VvRboh protein sequences with other plant homologs revealed several well-conserved functional domains (Figure 1). To date, all identified plant Rbohs have conserved binding sites for FAD, NADPH-ribose and NADPH-adenine, six TM domains with pairs of histidine residues in TM3 and 5, and two EF-hand, domains that are absent from the mammalian phagocyte pg91^phox^ protein [3]. EF-hands can bind Ca^2+^, which could account for the direct regulation of plant Rbohs by Ca^2+^[35]. Thus, the regulation of the plant proteins might be different from NADPH oxidases in mammalian phagocytes.

### 3.2. The Evolution of Rboh Proteins in Grape and *Arabidopsis* and Functional Prediction of *Vvrboh* Genes

Genomic comparison is a convenient and often effective way to transfer knowledge of genome structure and function gained from a well-studied taxon to a species where less information is available [36]. Thus, the predicted function of the *Vvrboh* genes might be suggested by a comparison with their respective orthologs in the model plant *Arabidopsis*, whose *rboh* genes have previously been characterized. A synteny analysis comparing the grape and *Arabidopsis* genomes showed that four paired *Vvrboh*-*Atrboh* genes located to syntenic genomic regions (Figure 4, Table S1) and the synteny analysis further indicated that these genes were derived from a common ancestor. The four orthologs in the syntenic map were also clustered together in the phylogenetic tree and may exhibit similar functions. *AT1G09090*, *AT5G47910*, *AT1G64060* and *AT4G11230*, correspond to *AtrbohB* and *AtrbohD*, *AtrbohF*, *AtrbohI*, respectively. Publicly available microarray data show that the expression of *AT4G11230* (*AtrbohI*) can be induced by anoxia, cycloheximide and norflurazone, and is only expressed in the root elongation zone [24]. *AtrbohB* is primarily expressed in germinating seeds and knocking out the expression of this gene disrupts seed germination [37]. Finally, AtRbohD and AtRbohF function in pathogen responses and stomatal closure [8,27]. It is possible that the grape homologs of these four *Arabidopsis* proteins could be involved in similar functions; however, further experimental analyses are necessary to confirm this.

### 3.3. Spatial Expression Patterns of *Vvrboh* Genes in Various Grape Tissues

There are several similarities in the spatial expression models of *Vvrboh* genes and those from other plant species. The AtRbohE, HvRbohE, and VvRbohE proteins are closely related and this is also reflected by their presence within the same phylogenetic group (IV) (Figure 2). *AtrbohE* is expressed preferentially in roots and seed tissues [24], and the barley gene *HvrbohE* is strongly expressed in roots, head and coleoptile tissues [21], while the closely related *VvrbohE* is highly expressed in roots, ovules and inflorescences. Members of groups I, III and V also appear to have similar expression patterns to each other. Torres, Onouchi, Hamada, Machida, Hammond-Kosack and Jones [9] as well as Sagi and Fluhr [24] reported that *AtrbohF* was expressed in all tested tissues/organs, and *HvrbohF1* an *HvrbohF2* were also constitutively expressed in all the tissues/organs examined [21]. This is similar to the expression patterns observed for *VvrbohA. HvrbohB1* and *HvrbohB2* are expressed in all tissues [21], as is *VvrbohB*, *VvrbohD* and *AtrbohD*. It therefore seems that members of individual *rboh* groups have similar expression signatures, again suggesting that there may be conserved functionality amongst members of the same groups.

### 3.4. *Vvrboh* Genes Respond to a Range of Biological Stresses

It has been reported that plant Rbohs mediate a wide range of responses to stimuli such as abiotic stress and development cues [3]. Several groups have reported that *rboh* genes are transcriptionally up-regulated by pathogens or fungal elicitors [11,15,16,38]. For example, *AtrbohD* and *AtrbohF* are required for accumulation of ROS during plant defense responses and studies of *Arabidopsis* mutants lacking functional *AtrbohD* and *AtrbohF* showed that *AtrbohD* is responsible for nearly all the ROS produced in response to avirulent bacterial or oomycete pathogens [27]. *NtrbohD* is responsible for ROS production after treatment of tobacco cells with the fungal elicitor cryptogein [15] and experiments using virus-induced gene silencing (VIGS) indicated that *NbrbohA* and *NbrbohB* are required for ROS accumulation and for resistance to *Phytophthora* [14]. *StrbohA* and *StrbohB* were induced by hyphal wall components from *P. infestans*, arachidonic acid and SA in potato tubers [16] and it has also been shown that transient RNAi-mediated gene silencing of *HvrbohA* led to an increase of basal penetration resistance during the penetration process of the powdery mildew fungus *B. graminis* f. sp. *hordei* [20].

ROS that is generated by plant Rboh proteins has also been implicated in regulating abiotic stress responses. For example *AtrbohD* and *AtrbohF*, which are expressed in guard cells, are transcriptionally induced by ABA treatment [4], and *AtrbohD* and *AtrbohA* have been shown to be involved in salt stress responses [24]. Gene expression studies in rice showed that *OsNox8* (*OsrbohI*) expression was significantly stimulated by NaCl stress, while *OsNox1* (*OsrbohB*) and *OsNox9* (*OsrbohH*) were strongly up-regulated by drought stress [35]. In addition, salt stress reduced the levels of *OsNox1* transcripts, but had no effect on *OsNox9* expression. We found by qRT-PCR analysis that six *Vvrboh* genes showed differential expression in response to at least one abiotic stress (Figure 6A,B), indicating their putative important roles in protecting grape from abiotic stresses. It is well known that ABA plays a crucial role in plant responses to abiotic stress, such as drought, salinity, cold, and hypoxia, and we found that the expression level of *VvrbohD* was strongly decreased following ABA treatment and salt stress. In contrast, *VvrbohB* was strongly up-regulated by exogenous ABA, drought and salt treatments and we propose that ROS produced by *Vvrboh* genes contributes to the response to drought via the ABA signaling pathways. In support of this idea, Pei, Murata, Benning, Thomine, Klüsener, Allen, Grill and Schroeder [4] demonstrated that ABA-induced H_2_O_2_ production and the H_2_O_2_-activated Ca^2+^ channels are important mechanisms for ABA-induced stomatal closing. Here *VvrbohA* and *VvrbohC1* were significantly stimulated by both drought and salt stress, but not to ABA treatment. This may be because the expression of these *Vvrboh* genes is regulated by ABA-independent signaling pathways when subjected to drought and salt stresses. Indeed, stress-responsive genes have previously been proposed to be regulated by both ABA-dependent and ABA-independent signaling pathways Shinozaki and Yamaguchi-Shinozaki [39].

In the current study, qRT-PCR analysis showed that *VvrbohB*, *VvrbohC2* and *VvrbohD* expression was up-regulated after powdery mildew inoculation (Figure 6C). This corresponds well with the predicted functions of *VvrbohC2* based on the syntenic analysis, which showed that *VvrbohC2*-*AT5G47910* (*AtrbohD*) represent an ortholog pair (Figure 4). Taking together, these results indicate that *rboh* genes in group I (Figure 2), *VvrbohC2*, *VvrbohD*, *NbrbohB*, *NtrbohD* and *AtrbohD*, are involved in pathogen resistance. However, although the three *Vvrboh* genes (*VvrbohB*, -*C2* and -*D*) may participate in resistance against powdery mildew, more work is needed to confirm their functions. Another interesting finding was that *VvrbohH* showed down-regulated expression after powdery mildew inoculation, which is in contrast with studies of other *rboh* genes, such as *Arabidopsis AtrbohF*, a mutation in which results increased resistance to a weakly virulent strain of the oomycete *Peronospora parasitica* [27]. This supports the idea that different *Vvrboh* genes exhibit divergent responses to pathogens and maybe even respond differently to distinct pathogens.

SA is one of the most widely studied plant stress-signaling molecules and its role in plant resistance to pathogens and other stress factors is well documented [40,41]. SA and ROS have been proposed to be involved in a positive feedback loop that amplifies signals leading to defense responses and cell death, and so ROS-dependent cell death and the accumulation of SA are intimately associated [42]. The expression level of *VvrbohD* strongly increased from 3 to 24 h after SA treatment (Figure 6D), as did that of *VvrbohH*, indicating a role for both genes in the SA signaling pathway.

## 4. Experimental Section

### 4.1. Identification and Annotation of Grape Respiratory Burst Oxidase Homolog *(Vvrboh)* Genes

Grape *rboh* genes (*Vvrboh*) were identified in the Grape Genome Database (12X) (http://www.genoscope.cns.fr) using the Hidden Markov Model (HMM) profile of the EF-hand binding domain (pfam00036), NAD binding domain (pfam08030) and FAD-binding domain (pfam08022) obtained from Pfam (http://pfam.sanger.ac.uk/). Subsequently, protein, gene and virtual cDNA sequences were all retrieved from the Grape Genome Database (12X) (http://www.cns.fr/externe/GenomeBrowser/Vitis).

### 4.2. Amino Acid Sequence Alignment and Phylogenetic Analysis

The predicted VvRboh protein sequences were aligned with homologous sequences in the public databases using the ClustalX [43], and the alignments were edited using ESPrit 2.2-ENDscript 1.0 (http://espript.ibcp.fr/ESPript/cgi-bin/ESPript.cgi; [44]). Putative transmembrane domains (TM), EF-hands (EF) and conserved binding sites for flavin adenine dinucleotide (FAD), NADPH-ribose and NADPH-adenine were predicated using the SMART program (http://smart.embl-heidelberg.de) and TMpred (http://ch.embnet.org/software/TMPRED_form.html; [45]). A schematic representation of the VvRboh protein functional domains was made using the DOG 1.0 software (http://dog.biocuckoo.org; [46]) and phylogenetic trees were constructed using the MEGA 5.0 software (Arizona State University, Tempe, AZ, USA) with the neighbor-joining (NJ) method and the 1000 bootstrap test replicates [47].

### 4.3. Exon/Intron Structure Analysis of *Vvrboh* Genes

The exon/intron structures of the *Vvrboh* genes were determined based on alignments of their coding sequences with corresponding genomic sequences using the est2genome program [48]. A diagram of exon/intron structures was obtained using the online Gene Structure Display Server (GSDS: http://gsds.cbi.pku.edu.ch; [49]), which indicates both exon position and gene length.

### 4.4. Synteny Analysis

Synteny blocks within the grape genome and between the grape and *Arabidopsis* genomes were downloaded from the Plant Genome Duplication Database (http://chibba.agtec.uga.edu/duplication) and those containing *Vvrboh* gene sequences were identified. Visualization of blocks was performed with Circos as described by Krzywinski M *et al*. [50].

### 4.5. Targeting Signal Prediction

Targeting signals of the predicted VvRboh proteins was performed with the aid of PSORT (http://psort.hgc.jp/form.html) [51].

### 4.6. Plant Materials

Grape tissues/organs, including young roots, stems, leaves, and tendrils, as well as inflorescences at the time of flower opening, berries and ovules at 33 days after flowering, were harvested from eight year-old “Kyoho” (*V. labrusca* × *V. vinifera*) grapevines grown in the field. Two year-old “Kyoho” juvenile plants were used for high salt, drought stress, exogenous hormone treatments and powdery mildew (*Uncinula necator* (Schw.) Burr.) inoculation. Grapevines were grown in the grape germplasm resource orchard of Northwest A&F University, Yangling, China (34°20′ N, 108°24′ E).

### 4.7. Abiotic and Biotic Stress Treatments and Hormone Applications

For abiotic stress assays, two year-old “Kyoho” grape juvenile plants grown in pots were irrigated with 2 L 250 mM NaCl [52]. After treatment for 1, 3, 6, 12, 24 and 48 h, respectively, fully unfolded young leaves were sampled. For drought treatment, watering was withheld for up to 7 days from potted “Kyoho” plants grown in the field in June, until the leaves showed wilting [53]. Briefly, the fully expanded young leaves of the plants were harvested at 24, 48, 72, 96, 120, 144 and 168 h post treatment. For salt and drought treatments, plants watered every three days and grown under the same conditions were used as a control.

Hormone treatments were conducted by spraying young leaves with 100 μM salicylic acid (SA) [54] or 200 μM abscisic acid (ABA) [55] followed by sampling at 1, 3, 6, 12, 24 and 48 h post-treatment. Leaves sprayed with sterile distilled water at the same time points were used as a negative control. Pathogen treatment was carried out by inoculating young leaves of “Kyoho” with powdery mildew as previously described with some modification [56]. Prior to inoculation, leaves were sprayed with sterile water, and then harvested at 6, 12, 24, 48, 72 and 120 h post-inoculation. Control plants were sprayed with sterile water at the same time points and not inoculated. Each treatment included three biological replicates with leaves from three plants being pooled and snap-frozen in liquid nitrogen and stored at −80 °C until further use.

### 4.8. Quantitative RT-PCR Analysis

Total RNA from grapevine was extracted using the E.Z.N.A.^®^ Plant RNA Kit (Omega Bio-tek, Norcross, GA, USA, R6827-01), then 500 ng of total RNA was used for first-strand cDNA synthesis using PrimeScript™ RTase (TaKaRa Biotechnology, Dalian, China). Products from this reaction were diluted six times and stored at −40 °C. Quantitative RT-PCR was conducted in triplicate using SYBR green (TaKaRa Biotechnology, Dalian, China) and an IQ5 real time PCR machine (Bio-Rad, Hercules, CA, USA). The conditions for the reactions were 95 °C for 30 s, 40 cycles of 95 °C for 5 s and 60 °C for 30 s, followed by a melt-curve analysis of 95 °C for 15 s and a constant increase from 60 to 95 °C. Grapevine *Actin1* (GenBank accession number AY680701) was amplified as an internal control. Primers used for qRT-PCR are listed in Table S2. Besides the technical replicates, three independent biological replicates were also analyzed. Relative expression levels were analyzed with the IQ5 software (Bio-Rad, Hercules, CA, USA) using the Normalized Expression method and a student *t*-test performed using the SPSS software (SPSS 17.0^®^, Chicago, IL, USA).

## 5. Conclusions

In the present study, seven grape *Vvrboh* genes were identified and partially characterized, thereby contributing to the growing knowledge of plant homologs of the human phagocyte *gp91^phox^* gene and its relatives. Syntenic and phylogenetic analysis helped to refine the resolution of the relationship between Rboh family members in various plant species and suggested possible functional roles for the grape Rbohs. The expression patterns of *Vvrboh* genes varied under different treatments, indicating diverse functions in plant stress responses. Future work will focus on functional analysis of the corresponding proteins.

## Supplementary Information



## Figures and Tables

**Table 1.** *RBOH* genes in grape.

Gene ID	Gene Locus ID	Accession No.	Putative function	Chromosome	Start	End	Predicted gene length (kb)	Predicted ORF length (bp)
*VvrbohA*	GSVIVT01019429001	XP_002277529.1	plasma membrane	chr2	621477	631791	10.310	2769
*VvrbohB*	GSVIVT01031128001	XP_002283888.1	chloroplast thylakoid membrane	chr14	1892425	1897786	5.362	2622
*VvrbohC1*	GSVIVT01014350001	XP_002282296.2	plasma membrane	chr19	2924506	2929619	5.114	2523
*VvrbohC2*	GSVIVT01001122001	XP_002268604.1	chloroplast thylakoid membrane	chr1	22798594	22814521	15.930	2484
*VvrbohD*	GSVIVT01001123001	XP_002268641.1	plasma membrane	chr1	22815076	22819209	4.134	2721
*VvrbohE*	GSVIVT01015025001	XP_002277540.1	chloroplast thylakoid membrane	chr11	542212	547987	5.776	2754
*VvrbohH*	GSVIVT01025074001	XP_002281695.1	chloroplast thylakoid membrane	chr6	4762503	4766209	3.707	2559

## Abbreviations

ABA: abscisic acid
At: *Arabidopsis thaliana*
HR: hypersensitive response
ORF: open reading frame
qRT-PCR: quantitative reverse transcription PCR
SA: salicylic acid
Vv: *Vitis vinifera*
