# Supplementary material for: Genome-Wide Analysis of Respiratory Burst Oxidase Homologs in Grape (Vitis vinifera L.)
Source: Int J Mol Sci. 2013 Dec 12;14(12):24169–86. doi: 10.3390/ijms141224169 (PMC3876103; doi:10.3390/ijms141224169)
Supplement: Supplementary file 1 [file ijms-14-24169-s001.pdf]

Supplementary Information

Table S1. Synteny blocks of rboh genes between the grape and *Arabidopsis* genomes.

ID	Region 1 (Ath)		Region2 (Grape)				Gene in the synteny region		
	Chr	Start	Stop	Chr	Start	Stop	Gene 1	Gene 2	Gene 2 name
73	Chr1	2879642	3100357	chr14	2914570	115071	AT1G09090	GSVIVT01031128001	*VvrbohB*
153	Chr1	23719783	23949751	chr2	433948	1583057	AT1G64060	GSVIVT01019429001	*VvrbohA*
573	Chr4	6820033	6940539	chr2	779587	213715	AT4G11230	GSVIVT01019429001	*VvrbohA*
621	Chr5	19381519	19407400	chr1	22982647	22715106	AT5G47910	GSVIVT01001122001	*VvrbohC2*

Table S2. Primers used in this study.

Primer name	Sequence (5'–3')
VvrbohA	F: TTGTGTCTGGCACCAGGGTTAG<br>R: TGCATTAGCGTGCTTGGTAGCTG
VvrbohB	F: CTTACCACCACCTACTCCTATCT<br>R: AAGAACGGCTGTCTACTTGTG
VvrbohC1	F: ACCTCAATCACGCCAAGAAGGG<br>R: ACCTCAATCACGCCAAGAAGGG
VvrbohC2	F: GCATCCTGATAGTCGAGTTGGC<br>R: TCCAAAGCTAGTTGCCGTAGATCC
VvrbohD	F: CCACAGTAGAACACTCCAACTC<br>R: GGTGTCAGGAACTCGTGTAAA
VvrbohE	F: TCCAAGCTATCAAGCATGCGAAAC<br>R: GCCTTGCAAAGTGTGTCCTTACTC
VvrbohH	F: GACTACTTGAGCGTCCACATT<br>R: CGGGAGGTTTGGTCATGTATAG
Actin1	F: GAT TCT GGT GAT GGT GTG AGT<br>R: GAC AAT TTC CCG TTC AGC AGT

© 2013 by the authors; licensee MDPI, Basel, Switzerland. This article is an open access article distributed under the terms and conditions of the Creative Commons Attribution license (http://creativecommons.org/licenses/by/3.0/).
